# Dyslipidemia in Adults with Type 2 Diabetes in a Rural Community in Ganadougou, Mali: A Cross-Sectional Study

**DOI:** 10.4236/jdm.2024.142012

**Published:** 2024-05-31

**Authors:** Abdoulaye Diawara, Djibril Mamadou Coulibaly, Drissa Kone, Mama A. Traore, Drissa Konaté, Dicko S. Bazi, Oumar Kassogue, Djeneba Sylla, Fatoumata Gniné Fofana, Oudou Diabaté, Mariam Traore, Ibrahim Antoine Nieantao, Kaly Keїta, Mamadou Diarra, Olivia Smith, Jian Li, Cheickna Cisse, Talib Yusuf Abbas, Crystal Zheng, Segun Fatumo, Kassim Traore, Mamadou Wele, Mahamadou Diakité, Seydou O. Doumbia, Jeffrey G. Shaffer

**Affiliations:** 1 University of Sciences, Techniques and Technologies of Bamako, Bamako, Mali; 2 National Federation of Community Health Associations, Bamako, Mali; 3 Department of Tropical Medicine, Medical Microbiology, & Pharmacology, John A. Burns School of Medicine, University of Hawaii Manoa, Honolulu, USA; 4 Department Biostatistics and Data Science, School of Public Health and Tropical Medicine, Tulane University, New Orleans, USA; 5 Department of Biotechnology and Computer Science, Burhani College, Mazgaon, Mumbai, India; 6 School of Medicine, Tulane University, New Orleans, USA; 7 Medical Research Council, Uganda Virus Research Institute, Entebbe, Uganda; 8 London School of Hygiene and Tropical Medicine, London, UK; 9 Departement of Biochemistry and Genetics Duquesne, University College of Medicine, Pittsburgh, USA

**Keywords:** Cholesterol, Cross-Sectional Study, Dyslipidemia, Lipids, Mali, Type 2 Diabetes

## Abstract

Dyslipidemia is a disorder where abnormally lipid concentrations circulate in the bloodstream. The disorder is common in type 2 diabetics (T2D) and is linked with T2D comorbidities, particularly cardiovascular disease. Dyslipidemia in T2D is typically characterized by elevated plasma triglyceride and low high-density lipoprotein cholesterol (HDL-C) levels. There is a significant gap in the literature regarding dyslipidemia in rural parts of Africa, where lipid profiles may not be captured through routine surveillance. This study aimed to characterize the prevalence and demo-graphic profile of dyslipidemia in T2D in the rural community of Ganadougou, Mali. We performed a cross-sectional study of 104 subjects with T2D in Ganadougou between November 2021 and March 2022. Demographic and lipid profiles were collected through cross-sectional surveys and serological analyses. The overall prevalence of dyslipidemia in T2D patients was 87.5% (91/104), which did not differ by sex (*P* = .368). High low-density lipoprotein cholesterol (LDL-C) was the most common lipid abnormality (78.9%, [82/104]). Dyslipidemia was associated with age and hypertension status (*P* = .013 and.036, respectively). High total and high LDL-C parameters were significantly associated with hypertension (*P* = .029 and .006, respectively). In low-resource settings such as rural Mali, there is a critical need to improve infrastructure for routine dyslipidemia screening to guide its prevention and intervention approaches. The high rates of dyslipidemia observed in Gandadougou, consistent with concomitant increases in cardiovascular diseases in Africa suggest that lipid profile assessments should be incorporated into routine medical care for T2D patients in African rural settings.

## 1. Introduction

Type 2 diabetes (T2D), a complex multifactorial disorder, is a rapidly emerging crisis in Africa [1]. In 2021, approximately 24 million adults 20 – 79 years old worldwide were living with T2D, which is expected to grow to 55 million persons by 2045 [2]. Approximately 416,000 deaths occurred in Africa in 2021 due to T2D, and it is estimated that 54% of those living with T2D were undiagnosed [2]. T2D is an established risk factor for dyslipidemia and occurs more frequently in T2D populations than non-T2D populations [3] [4]. Dyslipidemia is typically characterized according to the imbalance of lipids such as total cholesterol, low-density lipoprotein cholesterol (LDL-C), triglycerides (TG), and low high-density lipoprotein (HDL-C) [5]. Other risk factors associated with dyslipidemia include increased age, alcohol consumption, decreased vegetable consumption, low physical activity, and high waist circumference [6]. T2D is known to increase circulating free fatty acids and stimulate very low-density lipoprotein production [7]. T2D is also associated with elevated TG, increased LDL-C, and low HDL-C, with low HDL-C as the most regularly occurring lipid abnormality [8] [9]. Statin therapy and aggressive LDL-C control are recommended for persons with T2D [10] [11].

Lipid abnormalities are the primary link between T2D and cardiovascular disease (CVD), which may result in increased risk of stroke, sudden cardiac death, and myocardial infarction [3] [12]. Between 1990 and 2013, sub-Saharan West Africa was the only part of the world that experienced increases in CVD-related deaths that were not solely attributable to aging and population growth [13]. It is estimated that 80% of the world’s burden for CVD occurs in low-and middle-income countries (LMICs), while most of the studies on risk factors for CVD were performed in developed countries [14] [15]. Sub-Saharan Africa maintains the youngest age demographic associated with CVD deaths [16]. Unfortunately, in low-income countries, only 3% of rural communities have access to CVD preventive drugs [17].

The World Health Organization (WHO) recommends routine dyslipidemia screening based on age (over 40 years), smoking status, waist circumference, and presence of diabetes or hypertension [18]. While dyslipidemia is not widely reported in African countries, several recent studies have shown its high prevalence. Several of these studies linked dyslipidemia with age ≥ 40 years, less walking, elevated fasting blood glucose, and higher wealth index [19].

There are limited studies on dyslipidemia among T2D patients in African settings. In a systematic review of 177 studies at African sites, the prevalence of elevated total cholesterol concentrations, low HDL cholesterol concentrations, and elevated triglyceride concentrations were 34.4%, 39.5%, and 3.9%, respectively [20]. A recent study at public hospitals in Ethiopia among mostly urban T2D participants reported a dyslipidemia prevalence of 81.5%, with elevated TG being the most common lipid abnormality [21]. A study in South Africa among T2D participants showed dyslipidemia rates of 94.0% and 84.0% among males and females, respectively [22]. At least one study in Africa showed high dyslipidemia prevalence among T2D patients despite the use of lipid-lowering therapy [23]. High dyslipidemia rates in T2D participants have also been observed in northeastern parts of Africa [24]. In West Africa, a study in Nigeria reported that dyslipidemia occurred in 60.0% of its non-diabetic population and 89.0% of the diabetic population [25]. One study in Mali near its urban areas of Bamako, showed that 47.6% of T2D subjects had dyslipidemia [26].

In rural Mali, screening for dyslipidemia is not routinely performed in healthcare settings. To our knowledge, there are no studies reporting the prevalence of dyslipidemia in T2D populations in rural Mali. Such knowledge would provide a baseline for dyslipidemia occurrence in rural Mali, raise awareness about dyslipidemia in T2D populations, and guide healthcare policy in rural African settings. Given the lack of knowledge on dyslipidemia in rural Mali and recent evidence revealing the crisis of T2D in Africa, the investigators of this study sought to determine and report dyslipidemia prevalence and its associated demographic factors through a field study in Ganadougou, Mali.

## 2. Materials and Methods

### 2.1. Study Site

**Figure 1.**
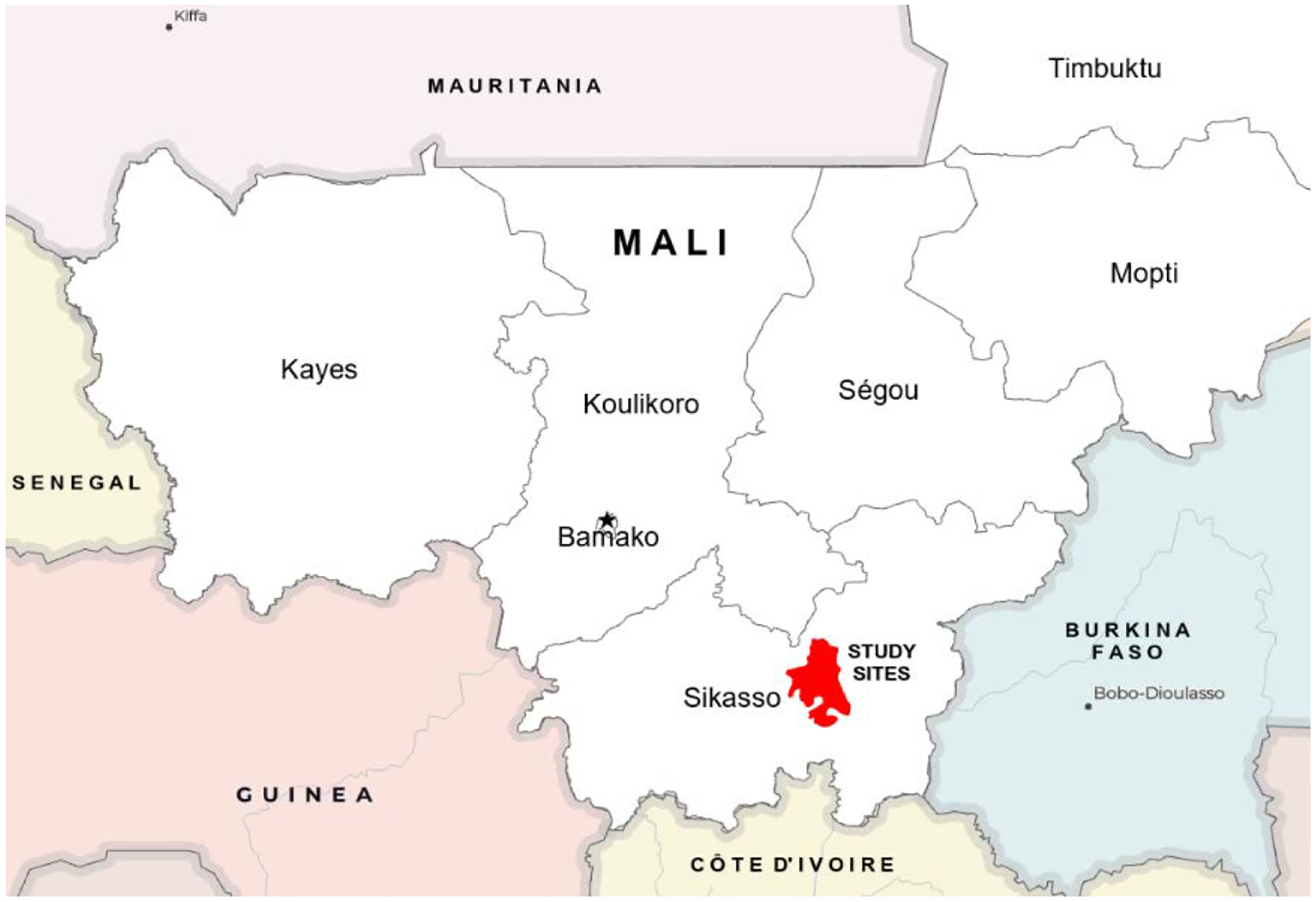
Geographical location of the study. The star represents Mali’s capital city of Bamako. The red-shaded region shows the selected study area. Participating municipalities included Nièna, Zanièna, Benkadi, and Finkolo-Ganadougou. Ganadougou is approximately 323 kilometers (201 miles) southeast of Bamako. Source and service layer credits for satellite imagery: Esri, DigitalGlobe, GeoEye, i-cubed, USDA FSA, USGS, AEX, Getmapping, Aerogrid, IGN, IGP, swisstopo, and the GIS User Community.

This was a prospective observational cross-sectional study carried out between November 2021 and March 2022, aiming at characterizing lipid profiles among residents in the rural commune of Ganadougou, Mali. Ganadougou is inhabited by the Fulani and Bambara populations. The community lies in the Sikasso Circle along the main freeway to Côte d’Ivoire. Ganadougou consists of 11 municipalities, and this study was performed in the municipalities of Nièna, Zanièna, Benkadi, and Finkolo-Ganadougou (Figure 1).

The Ganadougou community spans over 4000 square kilometers, and its capital city is Niéna. The community’s population is approximately 144,368, and its primary occupations are agriculture and animal husbandry. Ganadougou is approximately 323 kilometers (201 miles) southeast of Mali’s capital city of Bamako.

### 2.2. Study Design and Enrollment

Subject recruitment was drawn from an ongoing T2D campaign in the Ganadougou community that began in December 2020. This study was a cross-sectional design between November 2021 and March 2022. Inclusion criteria were age over 20 years, residency in the Ganadougou community, and confirmed T2D diagnosis. During this campaign, community residents were recruited and received free T2D rapid diagnostic test screening for study participation. A total of 1,417 participants were screened for T2D, and 104 persons with T2D (PWT2D) consented to participate. Cross-sectional surveys were used to collect data for all subjects, including demographic data, co-morbid conditions, and current medications for diabetics and hypertension. All PWT2D were referred for lipid profile assays at the Nièna Community Health Center. During this health center visit, blood pressure was measured using portable monitors, and hemoglobin A1c (HbA1c) and creatinine levels were measured. The blood sample was taken in a dry tube of sodium fluoride or sodium heparin lithium after 12 hours of fasting. After centrifugation, the appearance was identified and the serum was separated from the packed red cells and then assayed by enzymatic methods. Samples were analyzed using a KENZA 240TX blood chemistry analyzer (Biolabo Diagnostics, France).

### 2.3. Sample Size

Sample size was calculated based on the prevalence of dyslipidemia in the T2D population. The sample size formula applied the confidence interval for a single proportion as

$$n=\left(z_{1-\alpha }^{2}\cdot p\cdot q\right)/e^{2},$$

where *n* is the sample size, *z* is the normal percentile, α is the type I error rate, *p* is the estimated prevalence of dyslipidemia among PWT2D, *q* = 1 − *p*, and *e* is the margin of error. With *p* estimated at 63.8%, a 10% margin of error, and a type I error rate of 5%, the minimum required sample size was estimated as *n* = 89.

### 2.4. Definitions

The T2D cutoff for positive diagnosis was fasting plasma glucose level of 7.0 mmol/L (126 mg/dL) or higher [27]. Participants testing positive were invited to undergo a second rapid diagnostic test. Subjects were also classified as T2D subjects if they self-reported a previous T2D diagnosis on the study questionnaire. For purposes of this study, dyslipidemia was defined as the presence of one or more abnormal serum lipid concentration parameters. Abnormal serum lipid concentrations were defined as follows: serum total cholesterol (TC) level ≥ 5.2 mmol/L (≥200 mg/dL, hypercholesterolemia); HDL-C ≤ 1.3 mmol/L (≤50 mg/dL, females) and HDL-C ≤ 1.0 mmol/L (≤40 mg/dL, males); LDL-C ≥ 2.6 mmol/L (≥100 mg/dL); and serum TG ≥ 1.7 mmol/L (≥150 mg/dL, hypertriglyceridemia). Mixed dyslipidemia was considered as the presence of at least two abnormal lipid parameters. Hypertension was defined as systolic blood pressure of 140 mmHg or higher, diastolic blood pressure of at 90 mmHg or higher, or current use of antihypertensive drugs. The atherogenicity index was calculated as the LDL to HDL ratios, and ratios over 3.55 were considered as the presence of atherogenic risk. Blood creatinine levels above 106.1 μmol/L (1.2 mg/dL) were considered as hypercreatinemia. HbA1c values below 7.0% were considered as adequate glycemic control as per the American Diabetes Association guidelines [28]. Physical activity was classified in terms of occupational activites as: Light (limited physical activity, sitting office work, religious leader, and retirement activities), moderate (standing and walking, store work, and teaching activities), and active (walking, lifting, and heavy manual labor).

### 2.5. Statistical Analysis

Data were expressed as frequencies and proportions. Pearson’s chi-square tests were used to test hypotheses comparing proportions between comparison groups. Logistic regression models were applied to perform multiple comparison analyses. Data were analyzed using IBM SPSS Statistics for Windows, version 26 (version 26, IBM Corp., Armonk, NY) and the SAS System (version 9.4, SAS Institute, Inc., Cary, NC). The type I error threshold was set at 5%.

## 3. Results

### 3.1. Overall Dyslipidemia Prevalence and Participant Characteristics

The overall dyslipidemia rate was 87.5% (91/104). The most common lipid parameter abnormality was total cholesterol, where 66.3% (69/104) showed serum total cholesterol levels of 5.2 mmol/L (200 mg/dL) or higher (Table 1).

Notably, only 9.6% (10/104) of subjects had low HDL-c levels. Approximately 59.6% (62/104) of subjects had multiple lipid abnormalities. Among the 104 T2D participants, 76.9% (80/104) had HbA1c glycemic balance values of 7% or higher. Five of the 13 non-dyslipidemia participants (38.5%) had HbA1c levels of <7.0%.

Demographic, treatment, and comorbid factors known to be associated with T2D are shown by dyslipidemia status in Table 2.

Factors associated with dyslipidemia were age and hypertension (P = .013 and .036, respectively). Dyslipidemia status did not statistically differ according to sex, T2D treatment, T2D duration, hypercreatinemia status, glycemic balance, or physical activity.

### 3.2. Lipid Profiles

Study characteristics were classified by lipid parameters (high TC, low HDL-C, high LDL-C, high TG, and mixed dyslipidemia). None of the parameters statistically differed according to sex or age group. High TC and high LDL-c were associated with hypertension status (*P* = .029 and .006, respectively, Table 3).

Low HDL-c was significantly associated with HbA1c level (*P* = .003). Elevated atherogenicity index coincident with high TC, low HDL-C, high LDL-C, and mixed dyslipidemia (*P* = .008, <.001, .020, and .003, respectively). Abnormal lipid profiles were not significantly associated with T2D treatment, T2D duration, hypercreatinemia, or physical activity (*P* ≥ .050).

### 3.3. Glycemic Balance by Treatment Type

**Figure 2.**
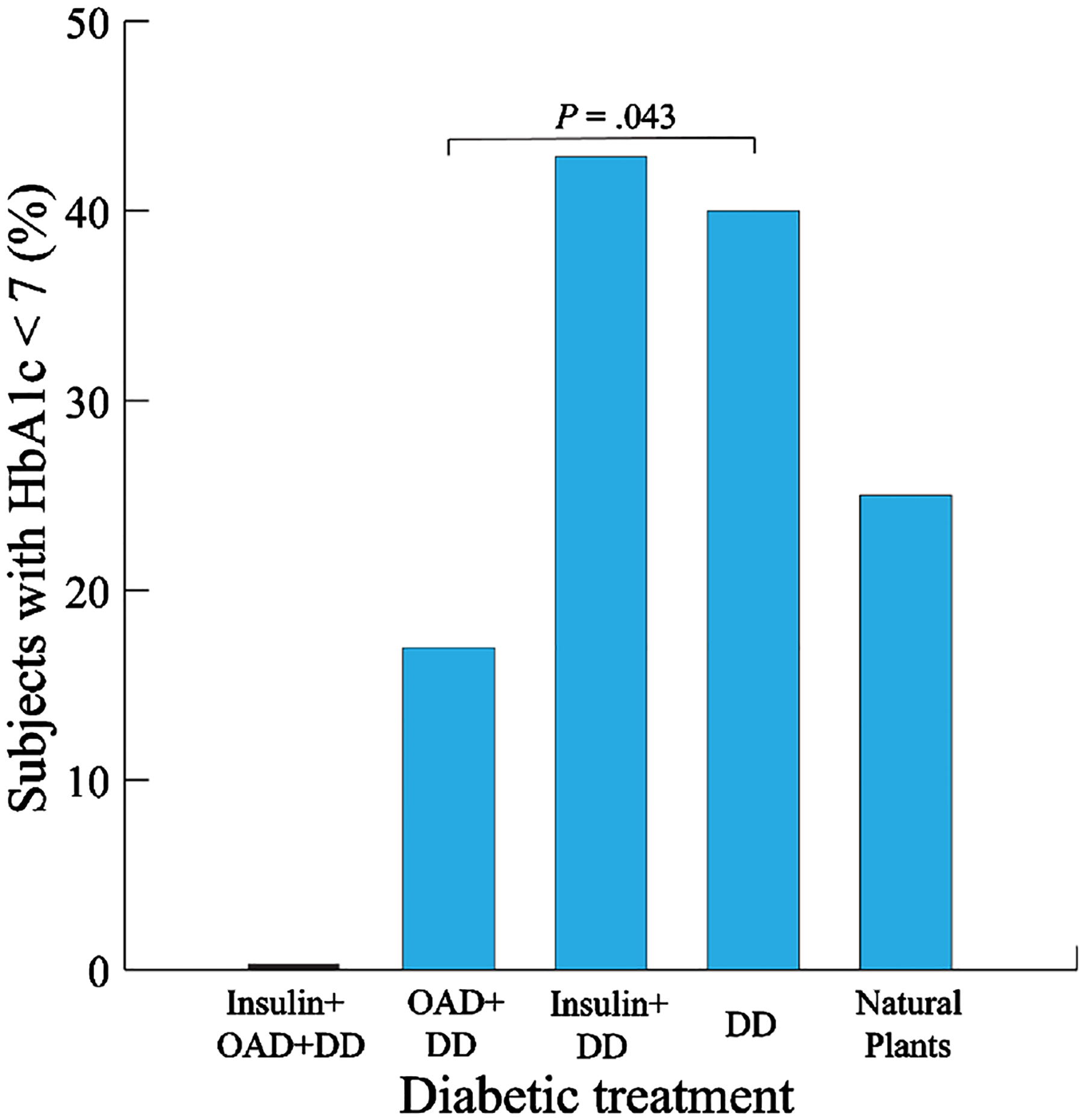
Glycemic balance by treatment type. DD = diabetic diet, OAD = oral antidiabetic drugs. Participants with DD alone were more likely than those receiving both OAD and DD to have an HbA1c < 7.0%.

Participants receiving insulin and DD were the most likely to have ideal T2D HbA1c levels (42.9% with HbA1c < 7). Those receiving DD alone were more likely than those with OAD+DD to have an HbA1c of less than seven (*P* = .043, Figure 2).

None of the eight subjects receiving insulin + OAD + DD had HbA1c values of less than seven. The lowest HbA1c values were observed for participants receiving OAD (0.0% and 17.0% for insulin plus OAD plus DD and OAD plus DD, respectively).

### 3.4. Hypertension by Sex

**Figure 3.**
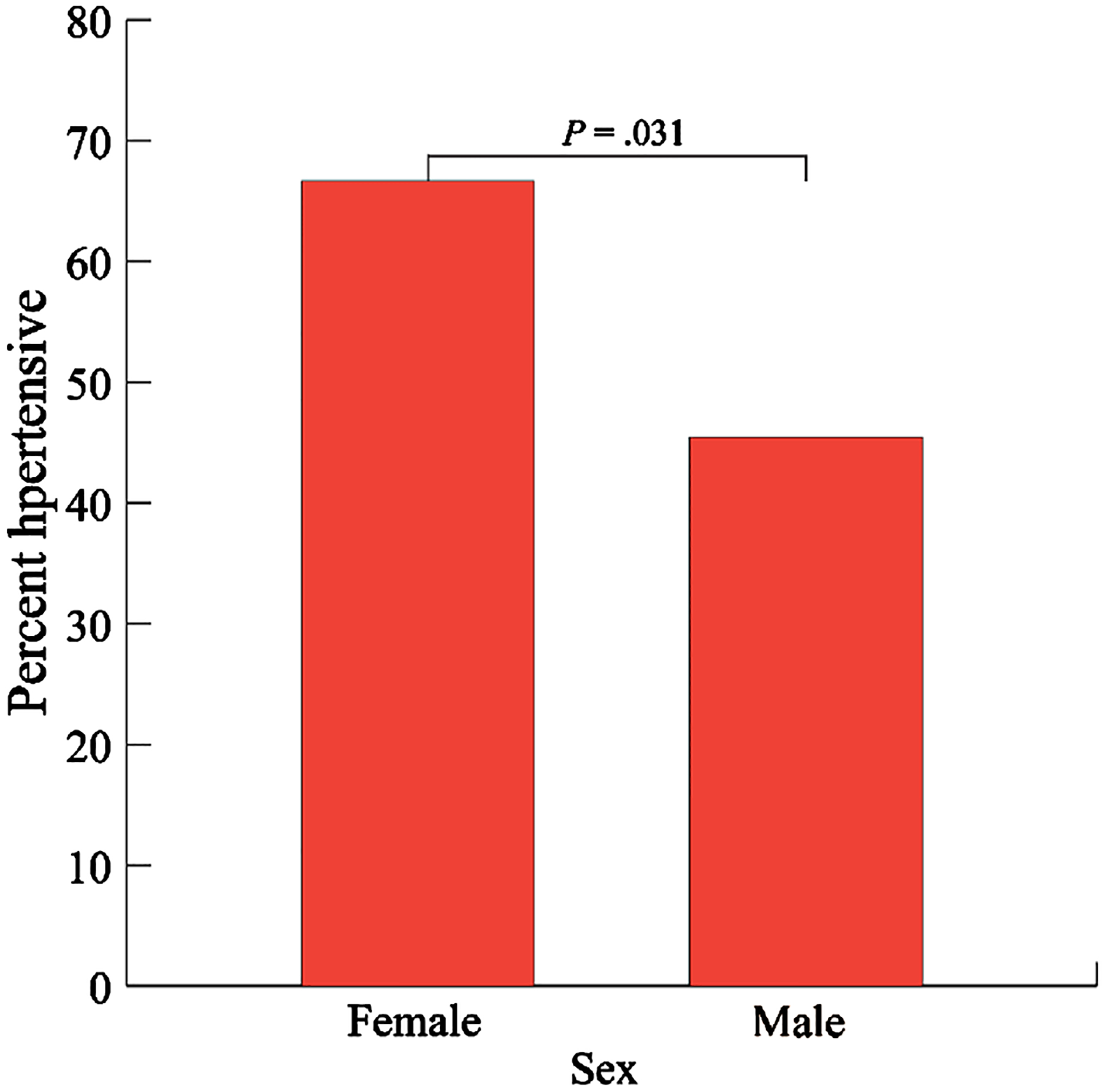
Hypertension by sex. Hypertension was defined as systolic blood pressure of at least 140 mmHg or diastolic blood pressure of at least 90 mmHg or current use of antihypertensive drugs. Hypertension rates were significantly higher among females than males (66.7% versus 45.5%, respectively, *P* = .031).

The overall percentage of T2D subjects with hypertension was 57.7% (60/104). Hypertension rates were significantly higher among females than males (66.7% versus 45.5%, respectively, *P* = .031, Figure 3).

The level of physical activity also differed between sex groups (*P* = .004, Table S1). Sex group did not differ by age group, treatment type, T2D duration, glycemic balance, or atherogenicity index (*P* = .732, .204, .938, .220, .464, and .522, respectively, Table S1).

## 4. Discussion

To our knowledge, this study is the first recent of its kind investigating dyslipidemia in rural Mali. Dyslipidemia prevalence was estimated as 87.5% among T2D subjects in the rural Malian community of Ganadougou. The high prevalence rates observed occurred across all age and sex groups. Glycemic balance was generally high among study subjects, suggesting that this group of T2D subjects was largely uncontrolled through treatment. While it is likely that uncontrolled diabetes in this rural community was impacted by the challenges for providing statin therapy in rural locations, other studies have shown that high dyslipidemia prevalence may persist among T2D populations receiving therapy [29]. The results in our study may provide a baseline pattern for lipid-lowering interventions such as statin therapy. The general lipid profiles here were similar to other studies, save for the unexpectedly normal HDL-c levels. It is possible that this finding is an artifact of the study’s sample size, and insufficient data were available for subgroup analyses as only four subjects reported low HDL-c. Some studies have described TC/HDL-cholesterol ratio as the single most predictive lipid factor [30]. In this study, HDL-c was normal for most subjects, which directly impacted these ratios.

Dyslipidemia has received little attention in West Africa. The majority of dyslipidemia studies are based on populations of European decent with limited studies based on African populations [31]. Among these African population-based studies, the majority are Northern African populations and Southern African populations. A PubMed key word search yielded only 20 hits through the key words “Mali” “dyslipidemia”, and only one of these hits corresponded to an article published since 2016. There are considerable challenges in dyslipidemia reporting in rural parts of Africa. First, screening is not typically incorporated into standard care. Incorporating information regarding dyslipidemia into lifestyle programs adapted for low- and middle-income countries such as Lifestyle Africa may offer a promising solution [32]. Dyslipidemia treatment sometimes only involves modest changes in monounsaturated and polyunsaturated fats (good fats) fat consumption coupled with weight loss. For this reason, telehealth and education campaigns may provide viable solutions for a. An integral treatment and education component would benefit from expressing quantitative lipid profiles in terms of their cardiovascular risk factors.

The importance of field studies in remote areas in Africa cannot be understated. Previous studies have shown that 79% - 100% of T2D subjects were initially diagnosed during cross-sectional surveys [33]. In one of our earlier studies, a majority (62.5%) of subjects diagnosed with T2D were unaware of their diabetic status [34]. These results suggest that carrying out field studies are beneficial beyond the research implications. While diagnostics for dyslipidemia are not widely available at many African field sites, potential reporting and monitoring solutions may consider the occurrence of related illnesses. For instance, many comorbidities related to dyslipidemia and T2D are bidirectional. The pathogenic relationship between T2D and hypertension is bidirectional [35].

Temporal trends for cardiovascular disease and T2D mortality rates have outpaced much of the developed world over the past three decades (8.3% to 13.1% and 1.0% to 2.0% between 1990 and 2020 for CVD and T2D, respectively, Figure S1). Because diabetes is one of its primary causes of dyslipidemia, it is likely that dyslipidemia occurrence has also increased. This study was performed in a rural area, but urbanization is spreading in Africa and cardiovascular risk profiles are reportedly worse in urban areas [36], which could suggest that this trend may increase in the absence of interventions. An initial first step toward raising awareness about dyslipidemia in PWT2D lies in raising awareness about T2D. It has been established that the nutritional transition is occurring in Africa [37]. A meta-analysis has shown a link between plant diets and lipid profiles [38]. Angassa *et al*. (2022) noted vegetable intake as a significant contributing factor for dyslipidemia in Ethiopia [6]. Longitudinal studies are needed to determine whether vegetable intake patterns are changing in Africa.

Studies have suggested that potentially all diabetics have abnormal lipid profiles [11], and thus diabetic therapy should consider lipid abnormalities. While point of diabetic care is a plausible approach, to our knowledge, none of the subjects in this study received adequate statin therapy. Previous researchers have suggested the use of fixed-dose combination therapy approaches to increase adherence [39]. This solution could perhaps partially address both cost for multiple treatments and adherence and distribution challenges. Others have suggested increased mobile healthcare programs [15]. Regardless of the approach, routine reporting and improving data systems in rural areas is needed. A better understanding health seeking behaviors related to dyslipidemia is also needed as good first steps.

Studies focusing on epigenetics are needed to complement and build on efforts from epidemiological studies. The LDL receptor protein and ATP-binding cassettes play key roles in lipid function [40]. Few investigations focus on these characteristics in Africa, studies are needed to complement data collected in developed countries. Within Africa, genetic associations differ by geographic location [31]. A natural progression for epidemiological studies for diabetic dyslipidemia lies in epigenetics. One example is a study which combined genetic and epigenetic analysis to understand the mechanism associating hepatic insulin resistance and non-alcoholic disease in (T2D) patients [41]. Epigenetics analyses have shown that environmental factors also have a crucial role in the development of T2D [42], which suggests that epidemiological studies may not necessarily generalize over geographic regions. It is worth mentioning that lipid imbalances in T2D subjects may be due to genetically determined disorders unrelated to glycemic balance or insulin resistance [43]. Genetic etiology of dyslipidemia is complicated, including both rare monogenic and complex polygenic disorders [44]. For example, mutations in LDLR (LDL receptor) and APOB (apolipoprotein B-100) genes may cause autosomal dominant hypercholesterolemia (a defect with severe life-long elevations in low-density lipoprotein-cholesterol) [45]; mutations in the LDLR adaptor protein 1 (LDLRAP1) gene cause autosomal recessive hypercholesterolemia [46]. Sex-specific genetic architecture has also been described previously [47]. For complex disorders, variants in genes with clear biological and clinical importance, such as LDLRAP1, SCARB1, and NPC1L1 have been identified, corresponding to approximately 25% - 30% of the genetic variance for various dyslipidemia traits [48]. It is likely that dyslipidemia among our subjects are a mixture of monogenic and polygenic disorders, which should be investigated in future works.

The primary limitations of this study were the convenience sampling strategy and the sample size. While the overall sample size provided adequate statistical power for general comparisons, the study was not powered for subgroup or multiple comparisons. Also, insulin storage was not always ideal which likely impacted the treatment results reported here.

## 5. Conclusions

Diabetic dyslipidemia was highly common in the rural T2D population studied here. This project was performed in a low-resource environment, where data systems were weak, and screening for dyslipidemia was either infrequent or did not previously occur. Multi-tiered strategies are needed for managing T2D that consider lipid status. Routine point-of-care screening of lipid profiles at rural public health units for adults at risk of T2D is needed.

## Supplementary Material

1

## Figures and Tables

**Table 1.** General lipid statuses among n = 104 subjects with type 2 diabetes.

Lipid status	n	%
Dyslipidemia	91	87.5
Abnormal total cholesterol	69	66.3
Abnormal HDL-c	11	10.6
Abnormal LDL-c	82	78.9
Abnormal triglyceride	57	45.2
Multi-dyslipidemia	73	70.2

*Note*. Abnormal serum lipid concentrations were defined as follows: serum total cholesterol (TC) level ≥ 5.2 mmol/L (≥200 mg/dL, hypercholesterolemia, high TC); HDL-C ≤ 1.3 (≤50 mg/dL, females, low HDL-C) and HDL-C ≤ 1.0 mmol/L (≤40 mg/dL, males, low HDL-C); LDL-C ≥ 2.6 mmol/L (≥100 mg/dL, high LDL-C); and serum TG ≥ 1.7 mmol/L (≥150 mg/dL, hypertriglyceridemia, high TG). Multi-dyslipidemia was defined by the presence of at least two abnormal lipid parameters.

**Table 2.** Characteristics of study subjects by dyslipidemia status.

Characteristic	Dyslipidemia Status^1^		*P*
	Dyslipidemia (n = 91)	Normal (n = 13)	
Sex			
Female	54 (59)	6 (46)	.368
Male	37 (41)	7 (54)	
Age group, years			
25 – 35	5 (5)	3 (23)	.013
36 – 45	12 (13)	5 (38)	
46 – 55	18 (20)	0 (0)	
56 – 65	35 (38)	3 (23)	
> 65	21 (23)	2 (15)	
Hypertension			
Yes	56 (62)	4 (31)	.036
No	35 (38)	9 (69)	
T2D/Hypertension Treatment			
Insulin + OAD + DD	8 (9)	0 (0)	.704
Insulin + DD	6 (7)	1 (8)	
OAD + DD	45 (49)	8 (62)	
DD only	17 (19)	3 (23)	
Natural plants	15 (16)	1 (8)	
T2D Duration, Years			
1 – 5	62 (68)	10 (77)	.800
6 – 10	21 (23)	2 (15)	
>10	8 (9)	1 (8)	
Hypercreatinemia^2^			
Yes	14 (15)	2 (15)	1.000
No	77 (85)	11 (85)	
Glycemic Balance, HbA1c, %^3^			
Optimal	19 (21)	5 (38)	.332
Elevated	38 (42)	5 (38)	
High	34 (37)	3 (23)	
Physical Activity			
Light	15 (16)	2 (15)	.975
Moderate	12 (13)	2 (15)	
Active	64 (70)	9 (69)	
Atherogenicity Index			
Elevated	17 (19)	0 (0)	.088
Normal	74 (81)	13 (100)	

*Note*. OAD = oral antidiabetic drugs, DD = diabetic diet.

1 Defined as the presence of one or more abnormal serum lipid concentration parameters.

2 Blood creatinine levels above 106.1 μmol/L (1.2 mg/dL) were considered as hypercreatinemia.

3 Measured as the percentage of glycated hemoglobin, <7 = optimal, 7 – 10 = elevated, >10 = high.

**Table 3.** Demographic and clinical characteristics by lipid profile serum parameters among n = 104 type 2 diabetics in Ganadougou, Mali, 2021.

Characteristic	n	High TC (N = 69)	Low HDL-C (N = 11)	High LDL-C (N = 82)	High TG (N = 57)	Mixed^1^ (N = 62)
Sex						
Female	60	44 (73)	9 (15)	51 (85)	32 (53)	46 (77)
Male	44	25 (57)	2 (5)	31 (70)	25 (57)	27 (61)
*P*		.078	.087	.073	.724	.092
Age Group, Years						
25 – 35	8	5 (63)	0 (0)	5 (63)	4 (50)	5 (63)
36 – 45	17	9 (53)	2 (12)	12 (71)	7 (41)	10 (59)
46 – 55	18	12 (67)	5 (28)	16 (89)	8 (44)	13 (72)
56 – 65	38	25 (66)	3 (8)	30 (79)	23 (61)	27 (71)
>65	23	18 (78)	1 (4)	19 (83)	15 (65)	18 (78)
*P*		.577	.098	.515	.457	.728
Hypertension						
Yes	60	45 (77)	4 (7)	53 (66)	37 (62)	46 (77)
No	44	24 (55)	7 (16)	29 (88)	20 (45)	27 (61)
*P*		.029	.130	.006	.101	.092
T2D Treatment						
Insulin + OAD + DD	8	6 (75)	1 (13)	8 (100)	4 (50)	6 (75)
Insulin + DD	7	4 (57)	1 (14)	6 (86)	2 (29)	5 (71)
OAD + DD	53	34 (64)	5 (9)	39 (74)	30 (57)	36 (68)
DD	20	13 (65)	1 (5)	16 (80)	12 (60)	14 (70)
Natural Plants	16	12 (75)	3 (19)	13 (81)	9 (56)	12 (75)
*P*		.878	.737	.510	.677	.982
T2D Duration, Years						
1 – 5	72	48 (67)	6 (8)	56 (78)	39 (54)	49 (68)
6 – 10	23	14 (61)	5 (22)	18 (78)	15 (65)	17 (74)
> 10	9	7 (78)	0 (0)	8 (89)	3 (33)	7 (78)
*P*		.657	.107	.742	.260	.757
Hypercreatinemia						
Yes	16	9 (56)	0 (0)	11 (69)	9 (56)	9 (56)
No	88	60 (68)	11 (13)	71 (81)	48 (55)	64 (73)
*P*		.353	.135	.282	.900	.185
HbA1c, %						
<7	24	14 (58)	0 (0)	18 (75)	10 (42)	14 (58)
7 – 10	43	33 (77)	2 (5)	36 (84)	27 (63)	34 (79)
>10	37	22 (59)	9 (24)	28 (76)	20 (54)	25 (68)
*P*		.169	.003	.592	.248	.187
Physical Activity						
Light	17	14 (82)	2 (12)	15 (88)	10 (59)	15 (88)
Moderate	14	10 (71)	1 (7)	12 (86)	5 (36)	10 (71)
Active	73	45 (62)	8 (11)	55 (75)	42 (58)	48 (66)
*P*		.242	.900	.400	.303	.188
Atherogenicity Index						
Elevated	17	16 (94)	6 (35)	17 (100)	13 (76)	17 (100)
Normal	87	53 (61)	5 (6)	65 (75)	44 (51)	56 (64)
*P*		.008	<.001	.020	.050	.003

*Note*. HbA1c: glycated hemoglobin, TC = total cholesterol, LDL = low-density lipoprotein cholesterol, HDL-C: high-density lipoprotein TG: triglycerides, OAD = oral antidiabetic drugs, DD = diabetic diet. Abnormal serum lipid concentrations were defined as follows: serum total cholesterol (TC) level ≥ 5.2 mmol/L (≥200 mg/dL, hypercholesterolemia, high TC); HDL-C ≤ 1.3 (≤50 mg/dL, females, low HDL-C) and HDL-C ≤ 1.0 mmol/L (≤40 mg/dL, males, low HDL-C); LDL-C ≥ 2.6 mmol/L (≥100 mg/dL, high LDL-C); and serum TG ≥ 1.7 mmol/L (≥150 mg/dL, hypertriglyceridemia, high TG). Mixed dyslipidemia was defined by the presence of at least two abnormal lipid parameters. Hypertension was defined as systolic blood pressure of at least 140 mmHg or diastolic blood pressure of at least 90 mmHg or current use of antihypertensive drugs.

1 Defined as the presence of at least two abnormal lipid parameters.

## Data Availability

Data supporting the results for this study are provided as tables within the article and as supplementary materials. 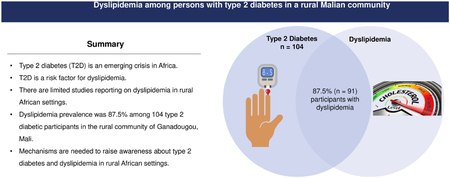 Dyslipidemia among persons with type 2 diabetes in a rural Malian community

## Abbreviations

BMI: body mass index
CVD: cardiovascular disease
DD: diabetic diet
HbA1c: glycated hemoglobin
HDL-C: high-density lipoprotein
IDF: International Diabetes Federation
LDL-C: low-density lipoprotein cholesterol
LMICs: low- and middle-income countries
OAD: oral antidiabetic drugs
OR: odds ratio
T2D: type 2 diabetes
TC: total cholesterol
TG: triglycerides
WHO: World Health Organization
