# Supplementary material for: Dyslipidemia in Adults with Type 2 Diabetes in a Rural Community in Ganadougou, Mali: A Cross-Sectional Study
Source: J Diabetes Mellitus. Author manuscript; Available in PMC 2024 Jun 27. (PMC11210374; doi:10.4236/jdm.2024.142012)
Supplement: 1 [file NIHMS2002022-supplement-1.pdf]

Supplementary Materials

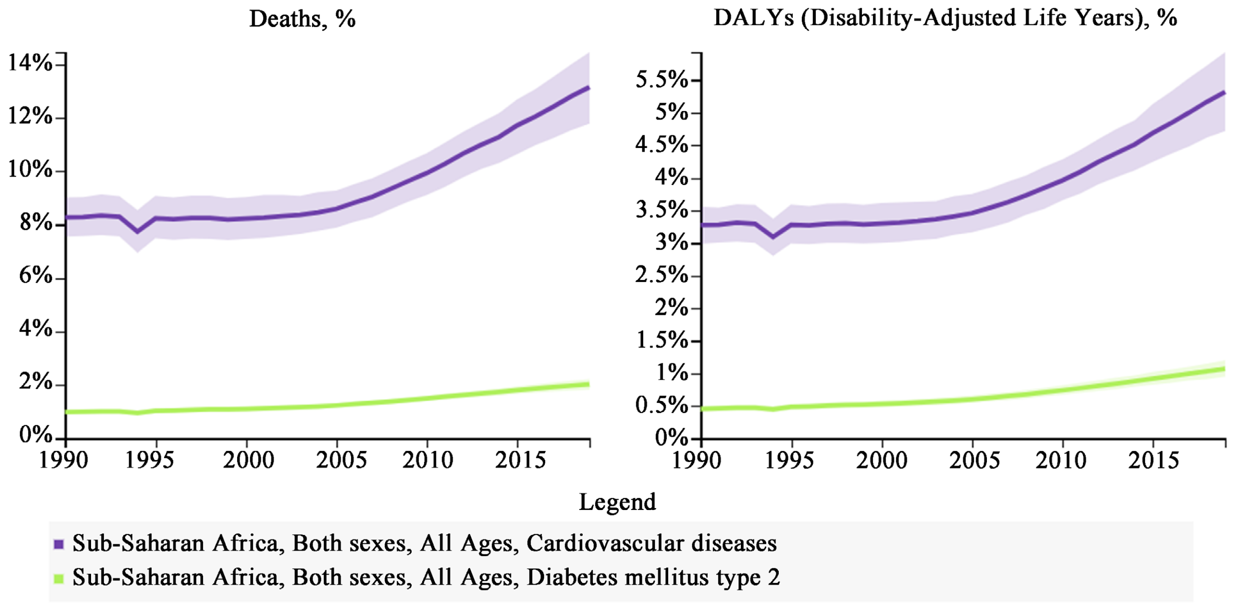

Figure S1. Temporal trends in cardiovascular disease and T2D in sub-Saharan Africa, 1990-2020. The purple and green lines correspond to the percent of overall deaths and disability adjusted life years for all cardiovascular diseases and type 2 diabetes, respectively. Between 2009 and 2019, deaths due to cardiovascular disease increased by approximately 57.8% and 100%, respectively. Source: 2019 Global Burden of Disease Study (https://ghdx.healthdata.org/gbd-results-tool).

Table S1. Study characteristics by sex.

Characteristic	Sex		*P*
	Female (n = 60)	Male (n = 44)	
Dyslipidemia[1]			
Yes	54 (90)	37 (84)	.368
No	6 (10)	7 (16)	
Age group			
25 - 35	5 (8)	3 (7)	.732
36 - 45	11 (18)	6 (14)	
46 - 55	12 (20)	6 (14)	
56 - 65	21 (35)	17 (39)	
>65	11 (18)	12 (27)	
Hypertension[2]			
Yes	40 (67)	20 (45)	.031
No	20 (33)	24 (55)	
T2D treatment			
OAD + DD + insulin	6 (10)	2 (5)	.204
OAD + DD	25 (42)	28 (64)	

Continued

Insulin + DD	5 (8)	2 (5)	
DD	12 (20)	8 (18)	
Natural plants	12 (20)	4 (9)	
T2D duration			
1 - 5	41 (68)	31 (70)	.938
6 - 10	14 (23)	9 (21)	
>10	5 (8)	4 (9)	
Hypercreatinemia			
Yes	7 (12)	9 (20)	.220
No	53 (88)	35 (80)	
HbA1c, %[3]			
< 7	14 (23)	10 (23)	.464
7 - 10	22 (37)	21 (48)	
>10	24 (40)	13 (29)	
Physical activity			
Light	8 (13)	9 (20)	.004
Moderate	3 (5)	11 (25)	
Active	49 (82)	24 (55)	
Atherogenicity index			
Normal	49 (82)	38 (86)	.522
Elevated	11 (18)	6 (14)	

Note. HbA1c = Glycated hemoglobin, T2D = type 2 diabetes, OAD = oral antidiabetic drugs, DD = diabetic diet. [1]Defined as the presence of one or more abnormal serum lipid concentration parameters. [2]Defined as systolic blood pressure of at least 140 mmHg or diastolic blood pressure of at least 90 mmHg or current use of antihypertensive drugs. [3]Measured as the percentage of glycated hemoglobin, <7 = optimal, 7 - 10 = elevated, >10 = high.
